# Diagnostic Pitfalls in Superior Mesenteric Artery Syndrome: A Case Report

**DOI:** 10.7759/cureus.97858

**Published:** 2025-11-26

**Authors:** Basma Alleelwa, Qazi Mohammad Waleed, Akanksha Soni, Mohammed Haitham Faeq Faeq, Aliyu O Olaniyi

**Affiliations:** 1 Acute Medical Unit, Stepping Hill Hospital, Stockport, GBR; 2 Internal Medicine, Mersey and West Lancashire Teaching Hospitals NHS Trust, Manchester, GBR; 3 Acute Medical Unit, Stockport NHS Foundation Trust, Stockport, GBR; 4 Critical Care, King's College Hospital NHS Foundation Trust, London, GBR; 5 Geriatrics, Stepping Hill Hospital, Manchester, GBR

**Keywords:** aorto-mesenteric angle, duodenal obstruction, gastrointestinal symptoms, superior mesenteric artery syndrome, weight loss

## Abstract

Superior mesenteric artery (SMA) syndrome is an uncommon yet clinically important cause of upper intestinal obstruction, occurring when the third segment of the duodenum is compressed between the superior mesenteric artery and the abdominal aorta, typically due to loss of the mesenteric fat pad that narrows the aorto-mesenteric angle and decreases the distance, most commonly following substantial weight loss. We present the case of a 52-year-old woman who developed persistent vomiting, diarrhoea, abdominal pain, and significant unintentional weight loss. Initial blood tests, stool studies, and endoscopic evaluation did not reveal any abnormalities. However, contrast-enhanced CT of the abdomen revealed significant narrowing of the space between the aorta and the superior mesenteric artery, along with a decreased vascular angle consistent with the diagnosis of SMA syndrome. She was managed conservatively with nutritional support, dietary modification, and postural therapy, resulting in gradual symptomatic improvement. This case highlights the diagnostic challenge of SMA syndrome and the importance of considering it in patients with unexplained weight loss and gastrointestinal obstruction symptoms, particularly when endoscopy is normal. Early recognition and radiological confirmation are crucial to guide appropriate management. Conservative treatment remains first-line, with surgery reserved for refractory cases. Maintaining a high index of suspicion for SMA syndrome is essential when evaluating patients with unexplained weight loss and persistent gastrointestinal symptoms.

## Introduction

Superior mesenteric artery (SMA) syndrome is an infrequent disorder that arises when the third part of the duodenum is constricted between the superior mesenteric artery and the aorta, leading to partial or complete duodenal obstruction [1]. The pathophysiology involves compression of the duodenum secondary to a decreased angle and shortened distance between the aorta and the superior mesenteric artery, changes that typically occur after substantial weight loss [2]. Patients commonly present with vague gastrointestinal complaints, including nausea (approximately 70%-77%), vomiting (65%-93%), abdominal pain (60%-83%), early satiety, and postprandial discomfort, which often hinder early recognition [1,3,4]. Diagnosis relies primarily on radiological evidence, as endoscopy is often unremarkable [5]. SMA syndrome is rare, with an estimated prevalence of 0.013% to 0.3% in the general population, although it may be underdiagnosed in high-risk groups such as those with rapid weight loss, spinal deformities, or prolonged immobilisation [6].

Normally, the aorto-mesenteric angle ranges from 28° to 65° with a distance of 10-34 mm, but in SMA syndrome, these values fall below 22° and 8 mm, respectively, leading to duodenal compression. The most frequent predisposing factor is rapid weight loss, which reduces the retroperitoneal fat pad and narrows the angle. Other risk factors include malnutrition, anorexia nervosa, chronic illness, spinal surgery, trauma, or prolonged bed rest. Symptoms may range from mild postprandial discomfort to severe vomiting and weight loss, often creating a vicious cycle of further narrowing. Diagnosis is best established with contrast-enhanced CT or CT angiography, which can confirm duodenal compression and exclude alternative causes.

## Case presentation

A 52-year-old woman attended the emergency department with an eight-week history of ongoing vomiting accompanied by diarrhoea. She reported an inability to tolerate oral intake, with vomiting occurring 30-60 minutes after meals, accompanied by abdominal cramps, bloating, and watery postprandial diarrhoea. She experienced unintentional weight loss of approximately 12.7 kg (two stone) during this period. She denied haematemesis, melena, rectal bleeding, fevers, or night sweats. Her medical history was notable for anxiety and depression, though she was not receiving regular pharmacological treatment, and a previous appendectomy. She reported consuming two lagers daily and regular use of vaping products.

On examination, she appeared cachectic, mildly dehydrated, and fatigued. Her vital signs showed a blood pressure of 130/90 mmHg, pulse of 112 bpm, afebrile status, and oxygen saturation of 99% on room air. She had a thin body habitus with a BMI of 17.4 kg/m². Abdominal examination demonstrated mild tenderness in the epigastric region, without evidence of organomegaly or peritoneal irritation, and bowel sounds were normal and active. There were no stigmata of chronic liver disease, lymphadenopathy, or peripheral oedema.

Routine laboratory tests, including full blood count, renal function, liver enzymes, C-reactive protein, and lipase, were within normal limits. Venous blood gas analysis showed no metabolic abnormalities (Table 1 ). Stool studies, including *Clostridioides difficile* toxin, stool culture, and faecal elastase, were negative.

**Table 1:** Summary of laboratory investigations. ALT: alanine aminotransferase, GFR: glomerular filtration rate, FT4: free thyroxine, GGT: gamma-glutamyl transferase, Hb: haemoglobin, Hct (PCV): haematocrit/packed cell volume, TSH: thyroid-stimulating hormone, Conc: concentration, Dist: distribution.

Test	Result	Reference
Serum total bilirubin	9 µmol/L	(1-21) µmol/L
Serum ALT	32 U/L	(0-33) U/L
Alkaline phosphatase	104 U/L	(20-130) U/L
Serum GGT	22 U/L	(9-36) U/L
Serum total protein	75 g/L	(60-80) g/L
Serum albumin	33 g/L	(34-47) g/L
Serum globulins	42 g/L	(22-42) g/L
Serum sodium	139 mmol/L	(133-146) mmol/L
Serum potassium	4.2 mmol/L	(3.5-5.3) mmol/L
Serum urea	2.7 mmol/L	(2.5-7.8) mmol/L
Serum creatinine	54 µmol/L	(44-97) µmol/L
Estimated GFR	> 90 mL/min	> 90 mL/min
White cell count	4.7 ×10^9^/L	(3.7-11.0) ×10^9^/L
Red cell count	4.19 ×10^12^/L	(3.80-5.80) ×10^12^/L
Haemoglobin	138 g/L	(115-165) g/L
Haematocrit (PCV)	0.42 L/L	(0.37-0.47) L/L
Mean cell volume	99.6 fL	(76.0-100.0) fL
Mean cell Hb	33.0 pg	(27.0-32.0) pg
Mean cell Hb Conc	331 g/L	(320-365) g/L
Red cell Dist width	13.00%	(11.5%-14.5%)
Platelet count	303 ×10^9^/L	(150-450) ×10^9^/L
Neutrophils	1.9 ×10^9^/L	(1.7-7.5) ×10^9^/L
Lymphocytes	1.4 ×10^9^/L	(1.0-4.5) ×10^9^/L
Monocytes	1.0 ×10^9^/L	(0.2-1.1) ×10^9^/L
Eosinophils	0.4 ×10^9^/L	(0.0-0.6) ×10^9^/L
Basophils	0.1 ×10^9^/L	(0.0-0.1) ×10^9^/L
Serum free thyroxine (FT4)	13.2 pmol/L	(10.0-22.1) pmol/L
Serum TSH	0.66 mU/L	(0.10-4.00) mU/L
Lipase	44 U/L	(12-53) U/L

**Figure 1:**
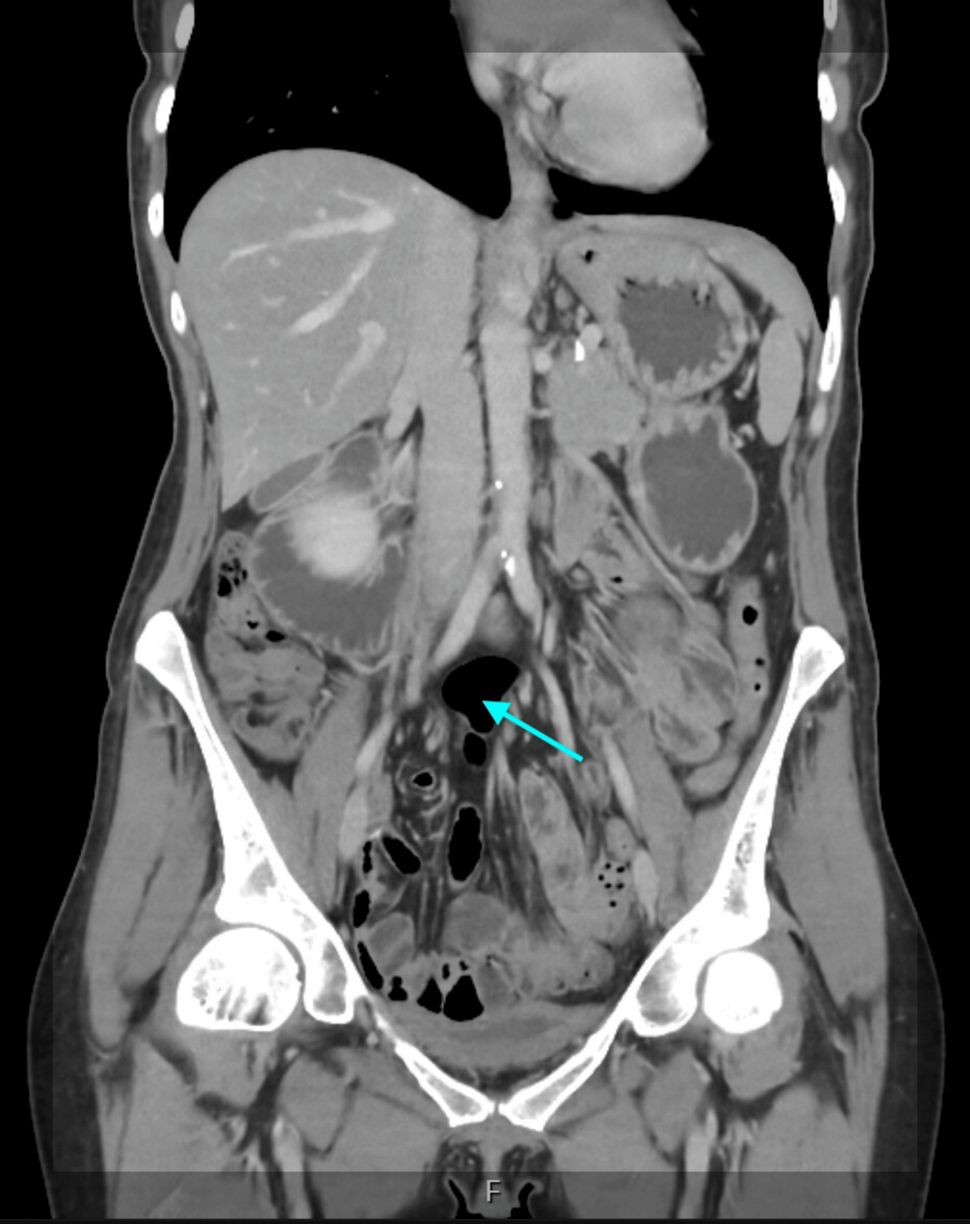
Coronal contrast-enhanced CT of the abdomen demonstrating a markedly distended stomach and proximal duodenum with abrupt narrowing of the third portion (arrow) as it traverses between the superior mesenteric artery and the aorta. This appearance reflects external vascular compression characteristic of superior mesenteric artery syndrome.

CT abdomen and pelvis with contrast revealed a distended stomach with fluid, dilatation of the first and second portions of the duodenum, and abrupt narrowing at the third portion where it crossed the SMA (Figure 1). The aorto-mesenteric angle was reduced to 16° (normal 28°-65°), and the aorto-mesenteric distance measured 5 mm (normal 10-34 mm). Minor inflammatory changes were seen in the small bowel loops.

Oesophagogastroduodenoscopy showed a normal oesophagus, stomach, and duodenum up to D2, with no obstructive lesions or ulceration. She was reviewed by gastroenterology and dietetics teams, and a conservative management plan was initiated. This included nutritional support with a high-calorie liquid diet, oral nutritional supplements, and advice to eat small, frequent meals. Postural therapy, including prone and left lateral decubitus positioning after meals, was recommended. Intravenous hydration and electrolyte replacement were provided initially, followed by oral fluids as tolerated. With close nutritional monitoring, her symptoms gradually improved, and she tolerated a soft diet. She was discharged with outpatient follow-up under gastroenterology and dietetics teams.

## Discussion

Superior mesenteric artery (SMA) syndrome is an uncommon cause of duodenal obstruction, accounting for an estimated 0.1%-0.3% of upper gastrointestinal obstruction cases [1]. It results from compression of the third portion of the duodenum between the abdominal aorta and the SMA due to a reduction in the aortomesenteric angle and distance [2]. Loss of the retroperitoneal fat pad, most often following rapid or substantial weight loss, is the most significant precipitating factor [4]. Additional risk factors include prolonged immobilisation, spinal deformities or corrective surgery, and prior abdominal operations [5].

A variety of clinical conditions contribute to SMA syndrome by altering aortomesenteric geometry or depleting the mesenteric fat cushion. Rapid weight loss, as seen in anorexia nervosa, malignancy-related cachexia, or chronic illness, can directly reduce the protective fat between the vessels and the duodenum. Corrective spinal surgery for scoliosis may increase vertebral height and modify anatomical relationships, thereby narrowing the aortomesenteric angle, particularly in individuals with low preoperative BMI. Trauma and prolonged immobilisation have also been implicated through mechanisms such as fat loss, postural changes, and tissue redistribution. These risk factors correlate with the commonly reported symptoms of nausea, vomiting, early satiety, and abdominal pain, underscoring the importance of recognising at-risk patients to prompt timely imaging and management [1,3]. The clinical presentation of SMA syndrome can be variable. Typical symptoms include nausea, vomiting, early satiety, abdominal pain, and bloating, often accompanied by weight loss and features of malnutrition [7]. Although diarrhoea is not a classic manifestation, it has been reported, potentially due to accelerated intestinal transit or malabsorption secondary to partial obstruction [8].

Diagnostic evaluation can be challenging. Endoscopy is typically normal and is primarily used to exclude alternative causes of upper gastrointestinal symptoms such as peptic ulcer disease or malignancy [9]. Because duodenal compression is external, the mucosa often appears normal; only in rare instances may endoscopy reveal proximal duodenal dilation, retained gastric contents, or extrinsic pulsatile compression. These findings are nonspecific and cannot confirm the diagnosis. Consequently, radiological imaging, particularly contrast-enhanced CT or CT angiography, remains the cornerstone of diagnosis, allowing direct visualisation of duodenal compression and precise measurement of the aortomesenteric angle and distance [10]. MRI and upper gastrointestinal contrast studies may provide complementary information.

Additional diagnostic tests help exclude alternative conditions that present with overlapping symptoms. Blood tests (e.g., full blood count, electrolytes, liver and pancreatic enzymes) can identify infectious, metabolic, or inflammatory disorders, while stool studies and nutrient panels may detect malabsorption syndromes such as coeliac disease or pancreatic insufficiency. Although important for differential diagnosis, these investigations are often normal in SMA syndrome, further emphasising the central role of radiological imaging in establishing a definitive diagnosis [1,2].

Management depends on symptom severity and underlying cause. Initial treatment is usually conservative and focuses on nutritional rehabilitation, dietary modification, and postural therapy to restore retroperitoneal fat and relieve duodenal compression [8]. Most patients improve with these measures; however, if symptoms persist despite adequate conservative therapy, surgical intervention, most commonly laparoscopic duodenojejunostomy, may be necessary [4]. Early recognition is crucial, as timely intervention can prevent disease progression and reduce the risk of complications.

## Conclusions

Superior mesenteric artery syndrome is a rare but important differential diagnosis in patients presenting with persistent vomiting, postprandial discomfort, and unexplained weight loss. Normal endoscopic findings should not exclude the condition, and CT imaging remains essential for diagnosis. Early recognition and nutritional rehabilitation are critical in management, with surgery considered for those unresponsive to conservative therapy. Clinicians should maintain a high index of suspicion for SMA syndrome in patients with unexplained gastrointestinal symptoms and significant weight loss, and it is advisable to specifically mention this possibility when requesting radiological imaging to ensure targeted assessment of the aorto-mesenteric angle and distance.
